# Systemic and Oral Health Parameters in Eutrophic and Overweight/Obese Adolescents: A Cross-Sectional Study

**DOI:** 10.3390/jpm13071073

**Published:** 2023-06-29

**Authors:** Martina Ferrillo, Dario Calafiore, Lorenzo Lippi, Antonella Petri, Alessandro Mastroianni, Leonzio Fortunato, Amerigo Giudice, Mario Migliario

**Affiliations:** 1 Dentistry Unit, Department of Surgical Sciences, University of Catanzaro “Magna Graecia”, 88100 Catanzaro, Italy; martina.ferrillo@unicz.it (M.F.); leo@unicz.it (L.F.); a.giudice@unicz.it (A.G.); 2 Physical Medicine and Rehabilitation Unit, Department of Neurosciences, ASST Carlo Poma, 46100 Mantova, Italy; dario.calafiore@gmail.com; 3 Physical Medicine and Rehabilitation Unit, Department of Health Sciences, University of Eastern Piedmont “A. Avogadro”, 28100 Novara, Italy; lorenzolippi.mt@gmail.com; 4 Translational Medicine, Dipartimento Attività Integrate Ricerca e Innovazione (DAIRI), Azienda Ospedaliera SS. Antonio e Biagio e Cesare Arrigo, 15121 Alessandria, Italy; 5 Pediatrics Unit, Department of Health Sciences, University of Eastern Piedmont “A. Avogadro”, 28100 Novara, Italy; antonella.petri@med.unipmn.it; 6 Dentistry Unit, Department of Clinical Sciences and Translational Medicine, University of Tor Vergata, 00133 Rome, Italy; dott.amastroianni@gmail.com; 7 Dentistry Unit, Department of Translational Medicine, University of Eastern Piedmont, 28100 Novara, Italy

**Keywords:** oral health, oral status, obesity, overweight, adolescents, dentistry, caries, prevention

## Abstract

To date, studies focusing on oral health in obese adolescents have provided controversial data. The aim of this cross-sectional study was to investigate systemic and oral health parameters in eutrophic and overweight/obese adolescents. In total, 100 adolescents, mean aged 13.33 ± 2.04 years, were divided into two groups: 59 overweight/obese adolescents in the study group (SG) and 41 eutrophic-weight adolescents in the control group (CG). Chi-squared and Fisher exact tests were performed to compare dichotomous and categorical variables between the two groups. The subjects in the SG (mean aged 13.21 ± 2.21) reported a body mass index (BMI) of 29.05 ± 4.09 kg/m^2^, corresponding to over 95° percentile for both genders, and the subjects in the CG (mean aged 13.49 ± 1.77) reported a BMI of 18.26 ± 4.81 kg/m^2^, corresponding to 25° percentile for both genders. In the SG, the serum level of 25-hydroxy-vitamin D was significantly lower (*p*-value < 0.001), whereas fasting blood glucose (*p* = 0.006), waist circumference, and hip circumference were significantly higher (*p*-value < 0.001). Plaque Index (PI), Plaque Control Record (PCR), Oral Hygiene Index (OHI), Gingival Index (GI), and Gingival bleeding index (GBI) depicted a significantly worse level of oral health in the SG. Moreover, the number of subjects with caries was significantly higher in the SG. Nutritional and physical activity status according to the Mediterranean Diet Quality Index for children and teenagers (KIDMED test) and the International Physical Activity Questionnaire (IPAQ-Adolescent) were reported to be significanlty better in the CG. In light of our results, obesity and poor oral health coexist in a cohort of adolescents. A screening of oral health status should be considered in obese subjects to focus resources on therapeutic interventions aiming at improving oral health.

## 1. Introduction

Childhood obesity can be considered as a worldwide public health problem and the prevalence has increased during the past decades [1]. It was estimated by the World Obesity Federation that there would be 206 million obese children/adolescents in 2025, and 254 million in 2030 [2]. Out of 43 European countries, Italy ranks as the 11th among overweight/obesity children aged 13 years and 20th among subjects aged 15 years [3]. In the pediatric age, gender-specific body mass index (BMI)-for-age percentile curves are usually used for the diagnosis of both overweight and obesity.

During childhood, subjects are considered overweight when the BMI ranges between the 85th and the 95th percentiles for age and gender, obese when the BMI is greater than the 95th percentile, and severely obese when the BMI is over the 99th percentile [4]. As the BMI defines only the total adiposity, other parameters are commonly used to assess body mass distribution, including the waist circumference (WC) and the waist/hip ratio (WHR) [1]. Central obesity is diagnosed when WC is higher than 102 cm in males and higher than 88 cm in females [5], and the 90th WC percentile was reported as the cutoff for adolescence [6]. On the other hand, WHR higher than 0.90 in males and higher than 0.85 in females defines abdominal obesity [5], and the cutoff was established at the 97th WHR percentile in male adolescents and at the 93rd–99th percentiles in female adolescents [7]. 

These anthropometric determinations were reported to be correlated with visceral adipose tissue, which has been shown to be able to secrete higher amounts of cytokines and hormones than the subcutaneous adipose tissue [8]. Moreover, overweight in childhood is associated with higher risk of systemic diseases, such as metabolic diseases, cardiovascular diseases, and cancers, compared to healthy-weight children [9,10]. On the other hand, the effect of obesity on oral health is less studied [11]. 

During the last decades the relationships between obesity and dental caries in children and adolescents have been investigated by epidemiological studies and systematic reviews [11,12,13,14,15,16]. Indeed, these two health problems share common risk factors, including dietary behavior, socioeconomic status, and parental education [12]. In this context, dental caries has been associated to the amount of consumption of sugar, and the relationship between sweetened beverages consumption and weight gain has been similarly explored in children [12,13]. Moreover, some studies have positively linked childhood obesity to periodontal diseases reporting plaque accumulation and increased gingival inflammation, but the causal relationships are unclear [11,14,15,16]. Periodontal diseases may lead to the destruction of soft and hard periodontal tissues through inflammatory mediators produced by bacterial and microbial biofilms, which can enter the systemic circulation and reach distant organs [17,18,19,20]. Moreover, the recent evidence has shown a potential link between periodontal diseases and some frequent systemic pathologies including diabetes, cardiovascular and respiratory diseases, cancer, and vitamin D deficiency or hypovitaminosis D [19,21,22,23,24,25,26,27,28]. 

However, the relationship between overweight/obesity and oral health specific to the pediatric population is still debated [29,30]. Van Dyke et al. demonstrated that obesity increases the systemic inflammatory load due to increased metabolic and immune parameters, increasing susceptibility to periodontal diseases [31]. A recent systematic review found a positive association between obesity and periodontitis in adults, showing how these two conditions might adversely affect each other [32]. Moreover, Suvan et al. suggested that obesity might play a role in both the onset and the progression of periodontal diseases [33]. In light of an increasing prevalence of overweight and obese children, it is of great importance to understand the early changes in the oral status of these patients.

Hence, the aim of this cross-sectional study was to investigate systemic and oral health parameters in eutrophic and overweight and obese adolescents.

## 2. Materials and Methods

### 2.1. Study Design and Participants

In this cross-sectional study, we recruited adolescents referred to the Pediatric Unit of the University Hospital “Maggiore della Carità”, Novara, Italy from January to December 2019. We included subjects with the following inclusion criteria: (a) age between 10 and 18 years old; (b) consent to undergo a specific dental evaluation. Exclusion criteria were: (a) suffering from genetic diseases (e.g., Prader Willi Syndrome, Down Syndrome, etc.); (b) suffering from metabolic diseases (e.g., Laurence-Biedl Syndrome, etc.); (c) suffering from endocrinological disease (Crushing Syndrome, etc.); (d) suffering from liver diseases. The study was conducted according to the “Strengthening the Reporting of Observational Studies in Epidemiology” (STROBE) and according to the Declaration of Helsinki. All participants signed informed consents and were free to withdraw from the study at any time.

The study participants, according to the auxo-grams proposed by Cacciari et al. [34], were divided into 2 groups: a study group including overweight and obese adolescents (over 95° percentile) and the control group including eutrophic adolescents (25° percentile). 

### 2.2. Clinical Assessment and Outcome Measures

Anamnestic and demographic data, including sex, age, weight, height, BMI, waist circumference and hip circumference, were collected. 

The following data regarding metabolic status were also collected: serum 25-hydroxy-vitamin D (25(OH)vit. D) (ng/mL), IGF-1 (ng/mL), and fasting blood glucose (mg/dL). Moreover, diastolic and systolic blood pressures were recorded. 

The diastolic and systolic blood pressure values (expressed in mmHg) were measured in a sitting position after at least 5 min of rest by means of a mercury sphygmomanometer. The calculation of the blood pressure percentiles was performed in accordance with the criteria suggested by the British Hypertension Society (National High Blood Pressure Education Program Working Group 2004) [35]. The subjects with systolic and diastolic blood pressure values below the 95th percentile were classified as normotensive and the subjects with systolic and diastolic blood pressure values above the 95th percentile as hypertensive [35]. 

Furthermore, all the enrolled subjects underwent a dental assessment at the Dentistry Unit of the University Hospital “Maggiore della Carità” of Novara to evaluate the following outcome measures: the Dental Formula, with annotation of number of permanent and deciduous teeth to assess dental caries and the dental treatment needs; the Plaque Index (PI), to evaluate the status of biofilm formation along the gingival margin, according to Löe and Silness [36]; Plaque Control Record (PCR) to evaluate the presence of plaque on the dental elements [37]; Oral Hygiene Index (OHI) to assess the presence of debris or stain and tartar on the dental elements [38]; Gingival Index (GI) to report gingival inflammation [36]; Gingival Bleeding Index (GBI) to define the percentage of gingival bleeding sites [39,40]. All indices were evaluated on the first molars and on the upper and lower central and lateral incisors. All the outcome measures were evaluated by the same observer. 

Moreover, 2 questionnaires evaluating nutrition and physical activity were administrated: the Mediterranean Diet Quality Index for children and teenagers (KIDMED test) [41] to estimate the adherence to the Mediterranean diet in children and adults (high: >8 points; moderate: 4–7 points; low: <3 points); the International Physical Activity Questionnaire (IPAQ-Adolescent) [32] to estimate the physical level activity (high: >7 h/week; between 3.5 and 7 h/week; low: <3.5 h/week). 

### 2.3. Statistical Analysis

Data management and statistical analyses were conducted according to a pre-specified statistical analytical plan and were performed using the STATA 14 (StataCorp LLC, College Station, TX, USA). The continuous variables were reported as means ± standard deviations. The Shapiro–Wilk test was performed to assess the distribution of all continuous data and the Wilcoxon rank sum test was used to compare the continuous variables when the data did not follow a normal distribution. Chi-squared and Fisher exact tests were performed to compare dichotomous and categorical variables between the two groups, with a *p*-value of 0.05 considered as statistically significant. 

## 3. Results

Out of 168 subjects screened according to the eligibility criteria, 100 subjects (51 females and 49 males, mean aged 13.33 ± 2.04 years) were included in the present study: 59 adolescents (mean aged 13.21 ± 2.21 years) in the SG, with a BMI of 29.05 ± 4.09 kg/m^2^, corresponding to over 95° percentile for both genders, and 41 adolescents (mean aged 13.49 ± 1.77 years) in the CG, with a BMI of 18.26 ± 4.81 kg/m^2^, corresponding to 25° percentile for both genders. Between-group statistically significant differences were reported for demographic variables: height, weight, waist circumference, and hip circumference (*p*-value < 0.001). Serum level of 25-hydroxy-vitamin D was lower in SG (17.40 ± 7.01 vs. 26.78 ± 9.13, *p*-value < 0.001), and fasting blood glucose was higher in SG (83.49 ± 11.53 vs. 77.46 ± 9.23; *p*-value = 0.006). Both systolic blood pressure (118.81 ± 12.02 vs. 105.49 ± 11.93, *p*-value < 0.0001) and diastolic blood pressure (78.25 ± 8.01 vs. 70.85 ± 9.55; *p*-value = 0.0001) were significantly higher in SG. Moreover, 39% of subjects (*n* = 23) in the SG and 10% of subjects (*n* = 4) in the CG were hypertensive, with statistically significant differences between groups (*p*-value = 0.001) (see Table 1 for further details).

In the SG, 68% (*n*= 40) had dental caries on permanent teeth; similarly, dental caries on deciduous teeth were found in 37% (*n* = 22) of SG. Data on oral health status are reported in Table 2. It was interesting to note that between-group significant differences were showed for all the analyzed indexes. 

Concerning the nutritional habits in the SG group, only three (5%) subjects had a high adherence to the Mediterranean diet and only four patients (7%) showed a high score of physical activity (further details are shown in Table 3).

## 4. Discussion

Obesity is a widespread condition that affects a growing number of children and adolescents worldwide [1,2,3]. Despite strong evidence supporting a strict relationship between obesity and periodontal diseases in adults [32], the influence of obesity on oral health status in adolescents has not been fully characterized. Thus, this cross-sectional study assessed oral health status in a cohort of obese adolescents, providing further evidence supporting the role of a screening in pediatric population focusing on patients with increased risk factors. 

To date, the World Health Organization (WHO) recommends an intake of free sugars lower than 10% of the total energy intake, and suggests an intake lower than 5% to reduce the risk of dental caries in both children and adults [42]. In order to prevent both obesity and caries, sugar should be taken as part of the main meal in a natural form (e.g., milk and fresh fruits) in order to replace fruit juices, smoothies, and other sweetened products [42]. 

It should be noted that several habits might be responsible for the higher incidence of dental caries in obese adolescents, and parental socioeconomic position (SEP), education, and income were reported as factors related to both obesity and oral health [43,44]. Indeed, it has been reported that a poor socioeconomic status and an inadequate parental education might be involved in the development of obesity, poor oral health, dental caries, and consequently a higher risk of oral surgery [45,46,47,48]. 

In this context, a recent study by Chen et al. [46] analyzed data from a large sample of 8446 families and showed that parents with higher education levels reported better oral health knowledge and more oral health care needs (including pit and fissure sealants), and that their children performed better oral hygiene home practices.

As reported by Martens et al. [49], obesity should be considered as a condition inducing a hyper-inflammatory state, which may lead to changes of T-lymphocyte and monocyte/macrophage characterized by higher pro-inflammatory cytokine production, including IL-1, TNF-a, IL-6, IL-8, C-reactive protein, and adipokines, which may increase gingival inflammation. Sex hormone serum levels might be affected by androgen disturbance in adipose tissues, which might be particularly important in obese pediatric patients. The results of the present study imply a significant between-group difference in terms of 25-hydroxy-vitamin D serum levels (SG: 17.40 ± 7.01 ng/mL vs. CG: 26.78 ± 9.13 ng/mL; *p*-value < 0.001). These results are particularly interesting considering the growing evidence supporting the role of vitamin D in oral health due to the widely documented interaction with inflammatory cytokines and other inflammation mediators [25,27,50]. In addition, it has been reported that vitamin D deficiency might lead to adipose tissue growth and body fat accumulation, with negative effects on skeletal muscle mass and function [51,52]. A recent systematic review with a meta-analysis of randomized controlled trials evaluated the efficacy of vitamin D supplementation on overweight and obese adolescents. The results showed that daily supplementation over 4000 IU/day of vitamin D could decrease the C-reactive protein and increase high-density lipoprotein levels [51]. Moreover, Gou et al. [51] highlighted the strict relationship between vitamin D serum level and insulin resistance, suggesting that supplementation should be recommend to prevent insulin resistance. Indeed, the results of the study indicate a significant improvement in homeostasis model assessment–insulin resistance (HOMA-IR) after vitamin D supplementation that implied improvement in insulin resistance, especially with a supplementation dose higher than 4000 IU/day [51]. Thus, vitamin D supplementation might have potential implications for overall well being in obese children and adolescents, including oral health status [53,54].

Moreover, the results of the present study report significant between-group differences in terms of fasting blood glucose (*p*-value < 0.001), systolic blood pressure, and diastolic blood pressure (*p*-value < 0.001 and *p*-value = 0.001, respectively), whereas for IGF-1, no statistically significant difference was shown. In greater detail, 39% of obese and overweight subjects and only 10% of normal weight patients were hypertensive. This was in line with Mallah et al., who reported arterial hypertension occurring in 10–30% of the obese children and less than 5% of the normal-weight subjects. Furthermore, the authors concluded that obesity was a risk factor for dyslipidemia, hypertension, metabolic syndrome, atrial fibrillation, and heart failure in children [55]. 

Moreover, obesity-associated immunological disorders were shown to induce modifications of the bacterial flora of the biofilm plaque, which may also predispose obese subjects to periodontal diseases [56]. In line with this, Modéer et al. analyzed samples of gingival crevicular fluid (GCF) to evaluate the levels of adiponectin, IL-β, plasminogen activator inhibitor-1 (PAI-1), TNF-α, and IL-8. The authors showed higher levels of IL-1 β (*p*-value < 0.001) and IL-8 (*p*-value < 0.002) in GCF from obese subjects compared with normal weight subjects [57], concluding that a close collaboration between dentists and pediatricians should be supported in the prevention and treatment of obesity.

In terms of oral-health parameters, our data highlight a significant between-group difference in terms of caries prevalence in permanent teeth (SG: 40 (68%) vs. CG: 14 (34%); *p* = 0.002) and in deciduous teeth (SG: 22 (37%) vs. CG: 6 (15%); *p* = 0.024), with approximately a double prevalence of caries in obese adolescents. Moreover, as PI and PCR may be considered as strongly dependent on the oral care hygiene routine, GI and GBI were also assessed to evaluate the signs of gingival inflammation. The two groups significantly differed in terms of PCR index (SG: 42.04 ± 0.18 vs. CG: 21.80 ± 0.14; *p*-value < 0.001) and GBI (SG: 2.86 ± 0.03 vs. CG: 1.17 ± 0.02; *p*-value < 0.001), thus suggesting that obese adolescents might have greater plaque accumulation. The relationship between obesity and periodontal inflammation might be related to a combination of metabolic and inflammatory profiles and a neglected attitude towards oral disease prevention, including self-hygiene procedures and periodical oral prevention recalls [48,49,56,57]. Our findings are in line with Modéer et al. [57], who showed more gingival inflammation (*p*-value < 0.001) and periodontal pockets deeper than 4 mm (*p*-value < 0.001) in obese compared to normal weight adolescents. Conversely, Vallogini et al. [58] showed lower PI and GBI in obese subjects compared to the non-obese group, which also showed better periodontal status in terms of probing depth. However, the obese subjects were enrolled in a pediatric hospital where they might have received dietary and hygiene counseling, whereas the normal weight participants were selected from a school. Contrariwise, a systematic review of epidemiological studies and controlled clinical trials found an undeniable pattern of increased risk of periodontitis in overweight and obese subjects, and the authors supposed that the development of insulin resistance related to the chronic inflammatory state and the oxidative stress might be implicated [59]. Strong evidence has underlined that obesity is characterized by increased blood proinflammatory cytokines (such as IL-1, IL-6, TNF-alpha) and oxidative stress activity [19,59]. On the other hand, proinflammatory states might interact with patients’ responses to oral antigens, with potential implications in host periodontal protection and immune response [60]. In addition, obesity in children and adolescents might promote oral colonization with unfavorable microbiota, including Firmicutes and Bacteroidetes sp., potentially promoting both oral inflammation and altered homeostasis [19,61,62]. However, to date, there are no clear indications about the role of oral dysbiosis in obese children, despite growing evidence related to this this topic in recent years [63,64]. In accordance, our data also show significant between-group differences in terms of GI, suggesting that obese children might be characterized by a higher risk of gingivitis. In line with this consideration, previous evidence suggests that gingivitis might be associated not only with systemic inflammation, but also with circulating sex hormones due to gender differences in terms of disease prevalence [61,65].

Lastly, significant differences were reported in terms of physical activity and nutritional status between groups (*p*-value < 0.001). Modifiable lifestyle factors, including physical activity and intake of high-fat and refined carbohydrates, have been shown to be responsible for the global increased prevalence of obesity and related health risks [66]. In this scenario, it is widely noted that lifestyle is a crucial factor affecting oral health [67]. In recent years, growing evidence has underlined that physical activity might be a key component of non-pharmacological interventions counteracting systemic inflammation with intriguing implications in oxidative stress tolerability and overall well-being [67,68,69]. Our results suggest that patients with poor physical activity levels might be characterized by a higher risk of both obesity and poor oral health, in accordance with previous studies in adults [70]. In addition, nutritional status should be considered as another key component of lifestyle intervention in a non-pharmacological approach targeting obesity and dental caries [12,43,44]. The Mediterranean diet is considered as one of the healthiest dietary models, related to lower rates of chronic disease morbidity and higher life expectancy [71]. Our findings suggest a lower level of MDQI in obese adolescents, and a nutritional intervention might represent a suitable option for obese patients not only to reduce the physical consequences of obesity and their impact on psychological well being [72], but also to reduce obesity’s implications in oral health so as to improve the comprehensive management of obese patients. 

Despite these positive results, we are aware that the present study is not free from limitations. First of all, the small sample size may not be representative of the Italian population, and the monocentric cross-sectional study design may not explain the multidimensional relationship between obesity and poor oral health. Moreover, all the oral health measurements were assessed on first molars and incisors, and a full-mouth evaluation was not performed. Furthermore, there is here a lack of a multivariable analysis, including gender differences, oral health status, and sociodemographic characteristics. Lastly, the most commonly used indicators of family socioeconomic position, education, and income were not analyzed. Therefore, further prospective studies are needed to understand the mechanisms underpinning the strict relationship between obesity and poor oral health to pave the way for a multicomponent intervention addressing poor oral health in obese children.

## 5. Conclusions

Taken together, the findings of this cross-sectional study show a high prevalence of dental caries, plaque on dental elements, and gingival inflammation in obese adolescents. As a result, a screening of oral health status should be considered in obese subjects to focus resources on therapeutic interventions aiming at improving oral health. Despite these considerations, further prospective studies are needed to characterize the effects of specific screening programs in obese children so as to better address the long term implications of poor oral status.

## Figures and Tables

**Table 1:** Demographic and clinical characteristics of children included in the study (*n* = 100).

	Over 95° Percentile (*n* = 59)	25° Percentile (*n* = 41)	*p*-Value
Age (years)	13.21 ± 2.21	13.49 ± 1.77	0.495
Sex			
*male (n/%)*	28 (47.5)	21 (51.2)	0.750
*female (n/%)*	31 (52.5)	20 (48.8)	
Weight (kg)	72.73 ± 16.54	40.52 ± 15.05	<0.001
Height (cm)	158.77 ± 9.34	147.32 ± 11.40	<0.001
BMI (kg/m^2^)	29.05 ± 4.09	18.26 ± 4.81	<0.001
Waist circumference (cm)	89.93 ± 9.88	63.44 ± 9.79	<0.001
*male*	92.45 ± 8.75	64.57 ± 7.85	<0.001
*female*	87.45 ± 9.78	62.85 ± 8.35	<0.001
Hip circumference (cm)	101.3 ± 11.48	75.41 ± 11.05	<0.001
*male*	98.32 ± 11.27	74.32 ± 10.57	<0.001
*female*	103.74 ± 10.65	75.98 ± 11.74	<0.001
(25(OH) vit. D) serum levels (ng/mL)	17.40 ± 7.01	26.78 ± 9.13	<0.001
IGF-1 (ng/mL)	302.87 ± 112.07	291.07 ± 164.24	0.670
Fasting blood glucose (mg/dL)	83.49 ± 11.53	77.46 ± 9.23	0.006
Systolic blood pressure (mmHg)	118.81 ± 12.02	105.49 ± 11.93	<0.001
Diastolic blood pressure (mmHg)	78.25 ± 8.01	70.85 ± 9.55	0.001
Hypertensive (*n*, %)	23 (39)	4 (10)	0.001

Continuous variables were reported as means ± standard deviations and categorical variables were reported as counts/percentages. Abbreviations: 25(OH)vit. D = 25-hydroxy-vitamin D; IGF 1 = insulin-like growth factor.

**Table 2:** Dental formula and oral hygiene status in children included in this study (*n* = 100).

	Over 95° Percentile (*n* = 59)	25° Percentile (*n* = 41)	*p*-Value
Permanent teeth (*n*)	23.88 ± 4.99	23.61 ± 5.06	0.7908
*Caries (n,%)*	*40 (68)*	*14 (34)*	*0.002*
Deciduous teeth (*n*)	1.20 ± 1.87	1.20 ± 1.87	0.982
*Caries (n,%)*	*22 (37)*	*6 (15)*	*0.024*
PI (*n*,%)			*<0.001*
*From 0% to 25%*	*0 (0)*	*0 (0)*	
*>25% and ≤50%*	*32 (54)*	*37 (90)*	
*>50% and ≤75%*	*26 (44)*	*4 (10)*	
*>75%*	*1 (2)*	*0 (0)*	
PCR	42.04 ± 0.18	21.80 ± 0.14	<0.001
OHI (*n*,%)			<0.001
*Optimal*	*0 (0)*	*0 (0)*	
*Good*	*28 (47)*	*33 (80)*	
*Sufficient*	*31 (53)*	*8 (20)*	
*Insufficient*	*0 (0)*	*0 (0)*	
GI (*n*,%)			*0.001*
*Optimal*	*33 (56)*	*36 (88)*	
*Good*	*26 (44)*	*5 (12)*	
*Sufficient*	*0 (0)*	*0 (0)*	
*Insufficient*	*0 (0)*	*0 (0)*	
GBI	2.86 ± 0.03	1.17 ± 0.02	0.003

Continuous variables were reported as means ± standard deviations and categorical variables were reported as counts/percentages. Wilcoxon rank sum test was performed as statistical analysis. Abbreviations: PI = Plaque Index; PCR = Plaque Control and Record; OHI, Oral Hygiene Index; GI = Gingival Index; GBI = Gingival Bleeding Index.

**Table 3:** Nutritional and physical activity status in children included in this study (*n* = 100).

	Over 95° Percentile (*n* = 59)	25° Percentile (*n* = 41)	*p*-Value
MDQI (*n*, %)			*<0.001*
*High*	*3 (5%)*	*10 (24)*	
*Moderate*	*18 (31)*	*27 (66)*	
*Low*	*38 (64)*	*4 (10)*	
IPAQ Adolescent (*n*, %)			*<0.001*
*High*	*4 (7)*	*17 (42)*	
*Moderate*	*16 (27)*	*21 (51)*	
*Low*	*39 (66)*	*3 (7)*	

Categorical variables here reported as counts/percentages. Abbreviations: MDQI = Mediterranean Diet Quality Index for children and teenagers; IPAQ Adolescent = Modified International Physical Activity modified.

## Data Availability

Dataset is available on request to the corresponding authors.
